# Knockout of MIMP protein in *lactobacillus plantarum* lost its regulation of intestinal permeability on NCM460 epithelial cells through the zonulin pathway

**DOI:** 10.1186/1471-230X-14-171

**Published:** 2014-10-03

**Authors:** Zhihua Liu, Liang Kang, Chao Li, Chao Tong, Meijin Huang, Xingwei Zhang, Nanqi Huang, Mary Pat Moyer, Huanlong Qin, Jianping Wang

**Affiliations:** Department of Colorectal Surgery, Gastrointestinal Institute of Sun Yat-Sen University, the Sixth Affiliated Hospital of Sun Yat-Sen University (Guangdong Gastrointestinal Hospital), 26 Yuancun Erheng Road, Guangzhou, Guangdong 510655 China; INCELL Corporation, San Antonio, Texas 78249 USA; Department of Surgery, Shanghai Jiao Tong University Affiliated Sixth People’s Hospital, Shanghai, 200233 China

**Keywords:** *Lactobacillus plantarum*, Micro integral membrane protein, Zonulin, Intestinal permeability

## Abstract

**Background:**

Previous studies indicated that the micro integral membrane protein located within the media place of the integral membrane protein of *Lactobacillus plantarum* CGMCC 1258 had protective effects against the intestinal epithelial injury. In our study, we mean to establish micro integral membrane protein -knockout *Lactobacillus plantarum* (LPKM) to investigate the change of its protective effects and verify the role of micro integral membrane protein on protection of normal intestinal barrier function.

**Methods:**

Binding assay and intestinal permeability were performed to verify the protective effects of micro integral membrane protein on intestinal permeability *in vitro* and *in vivo*. Molecular mechanism was also determined as the zonulin pathway. Clinical data were also collected for further verification of relationship between zonulin level and postoperative septicemia.

**Results:**

LPKM got decreased inhibition of EPEC adhesion to NCM460 cells. LPKM had lower ability to alleviate the decrease of intestinal permeability induced by enteropathogenic-e.coli, and prevent enteropathogenic-e.coli -induced increase of zonulin expression. Overexpression of zonulin lowered the intestinal permeability regulated by *Lactobacillus plantarum*. There was a positive correlation between zonulin level and postoperative septicemia. Therefore, micro integral membrane protein could be necessary for the protective effects of *Lactobacillus plantarum* on intestinal barrier.

**Conclusion:**

MIMP might be a positive factor for *Lactobacillus plantarum* to protect the intestinal epithelial cells from injury, which could be related to the zonulin pathway.

## Background

It has been proved that gut flora homeostasis in human intestine is mediated largely by probiotics, including *Lactobacillus plantarum* (LP) and other microorganisms [1–3]. LP can improve intestinal pathological disorders through the modulation of intestinal functions [2]. Therefore, as one of the best-characterized probiotic bacteria, LP can be selected in clinical trials assessing the prevention and treatment of intestinal disorders, such as the complications after surgical operation [2, 4, 5]. However, which components mostly contribute to the protective effects of LP still remains an interesting question to be further investigated [6].

Recently, the lactobacillus surface layer protein (SLP) has been raised as a key component mediating the protection conferred by LP to intestinal epithelial cells (IECs) [7, 8]. It is found that a 50-kDa protein extracted from the surface layer of lactobacillus could adhere to IECs and its mimic protein mucin [9]. SLP isolated from *Lactobacillus crispatus* showed the ability to inhibit adherence of enterotoxigenic *E. coli* to a synthetic basement membrane [10], and reduce both dextran flux and trans-epithelial electrical resistance (TER) [11]. SLP also reduced the number and rearrangement of α-actin foci, and attenuated bacterial colonization on IECs and pathogen-induced changes in cellular permeability [12]. Furthermore, soluble factors, including p75 and p40, extracted from *Lactobacillus rhamnosus* GG culture broth supernatant, showed protective effects on IECs, which was mediated by Akt pathway [13, 14]. Only a few studies have, however, investigated the structure and functions of SLP [8, 10, 15–18], due to the specific hydrophilic and hydrophobic properties and technical difficulties associated with SLP purification, thus limiting the investigation of SLP binding domains.

Zonulin has recently been discovered as a protein involved in tight junctions (TJ) between IECs [19]. Zonulin was originally discovered as the target of zonula occludens toxin, which has been reported with the increased gut permeability in the pathogenesis of coeliac disease [20]. Recent studies have indicated that zonulin got the regulative function of intestinal permeability and barrier [19, 21], suggesting that high expression of zonulin may cause the increase of intestinal permeability [22].

Our previous studies have demonstrated that LP was able to prevent IECs from injury induced by EPEC [23], regulate dendritic cells maturation and T-lymphocytes differentiation [24]. In addition, a protective role of TJ microstructure both *in vivo* and *in vitro* was also evidenced [25, 26]. In our study, the SLP (the integrated membrane protein, IMP) isolated from LP was extracted and purified [27]. To increase the specificity and valence of SLP, we further identified the small functional protein domain, the micro IMP (MIMP) [8], and confirmed the protective function of MIMP on IECs [16]. An additional study indicated that the molecular mechanism is related to the activation of protein kinase C-η and occludin phosphorylation [15]. Moreover, we identified the receptor of MIMP on NCM460 cells, and the mechanism of p38 MAPK signaling pathway [17], and showed that probiotics may exert its protective effects on the intestinal barrier through the zonulin pathway [28]. To verify the protective effects of MIMP, here we mean to establish MIMP-knockout LP (LPKM) and investigate the change of protective effects of LPKM on IECs against EPEC infection. Furthermore, we will also investigate the molecular signal transduction pathway during the interaction between LP and intestinal permeability.

## Methods

### NCM460 cells culture

NCM460 cells were purchased from INCELL Corporation (San Antonio, TX, USA) and cultured in M3 media supplemented with 10% Fetal bovine serum (FBS), 100 U/ml penicillin and 100 μg/ml streptomycin at 37°C in a 95% humidified atmosphere with 5% CO_2_. Cells were passaged at pre-confluent densities using 0.05% trypsin and 0.5 mm EDTA (Invitrogen, Carlsbad, CA) [17]. NCM460 cells were passaged 24 h before transfection.

### IL-10^-/-^ mice breeding and grouping

IL-10^-/-^ mice were generated on a wild-type 129 Sv/Ev genetic background, bred and raised in the animal facility at Shanghai Jiao Tong University, School of Medicine. Mice were housed under specific pathogen-free conditions until weaning (3 weeks) when they were moved to a conventional Animal Care Unit. Therefore, mice were housed in cages with a high-efficiency particulate air filter and fed with a standard mouse chow diet. IL-10^-/-^ mice at age of 3.5 weeks were randomized into three groups (n = 10 for each group), and were treated with oral gavage of milk alone or added with 1 × 10^9^ cfu/mL LP or LPKM. Wild-type (WT) mice were treated with oral gavage of milk (n = 10 for each group). The volume of gavage was 0.5 mL. Mice were treated up to the age of 17 weeks and then sacrificed by cervical dislocation. The Animal Care and Use Committee and the Ethics Committee of Shanghai Jiao Tong University approved the experimental protocol in compliance with the Helsinki Declaration (G2012007).

### Bacterial strains and culture conditions

The EPEC strain ATCC 43887 (O111:NM) (Shanghai Municipal Center for Disease Control and Prevention, Shanghai, China) was grown in DMEM at 37°C for 24 h. The LP strain (CGMCC 1258) was provided by the Institute of Bio-medicine, Shanghai Jiaoda Onlly Company Ltd, and cultured in MRS broth (Difco, Sparks, MD, USA) at 37°C. Quantification of bacteria was carried out by measuring the optical density at 600 nm using a Beckman DU-50 spectrophotometer to determine the colony forming units (CFU).

### MIMP targeting

The mutant strain LPKM was constructed with a deletion of MIMP using standard integration and excision methods, tools, and strains, as previously described [29, 30]. A pET32 deletion vector was constructed containing two targeting fragments, using the incision enzyme BglII and XhoI, which flank MIMP gene as previously described [8]. After a double crossover integration and excision event, LPKM was recovered harboring a 183-bp deletion of MIMP in the genome. PCR products over the IMP region in LPKM confirmed the loss of ~ 200 bp in the genes surrounding the deletion [31].

### Western blot analysis

Western blot was performed as previously described [17]. Visualization was performed using enhanced chemiluminescence according to the manufacturer’s instructions (ECLkit; Pierce, IL, USA).

### Binding assay of LP and the competitive inhibitive effect on MIMP

Cells were cultured as monolayers (~1 × 10^7^ for each well) and divided into experimental groups in triplicate as previously described [17]. In LP groups, LP (100 μL of 1.0 × 10^8^/mL) was added onto the monolayer of NCM460 cells simultaneously with EPEC infection. In LPKM groups, LPKM (100 μL of 1.0 × 10^8^/mL) was added onto the monolayer of NCM460 cells simultaneously with EPEC infection. In antibody groups, NCM460 cells were pre-incubated with the serum containing polyclonal anti-MIMP antibodies (100 μL of dilution 1:5000) prepared as previously described [8], prior to infection with EPEC which was simultaneously incubated with LP.

### Measurement of transepithelial electrical resistance (TER) and dextran permeability

The methods were described previously [17]. The intestinal epithelial monolayers were divided into five different experimental groups in triplicate.

### Measurement of the intestinal permeability and colonic damage in mice

The intestinal permeability was determined in treated or untreated IL-10^-/-^ and wild-type mice as previously described [17]. Final data were reported as either the fractional excretion (for sucralose) to determine the colonic permeability or a ratio of fractional excretion (for lactulose/mannitol) to quantify the small intestinal permeability. Fractional excretion was defined as the fraction of the gavaged dose recovered in the urine, and the ratio of fractional excretion was defined as the ratio of the fraction of the gavaged dose of lactulose recovered in the urine over the fraction of the gavaged dose of mannitol recovered in the urine.

### Ussing chamber assay to determine the intestinal permeability measurement in isolated mice colons

Treated or untreated IL-10^-/-^ and wild-type mice were sacrificed at 8 weeks and a Ussing chamber assay was performed as described previously [16, 32]. Tissue ion resistance (1/G, where G represents the conductance) was calculated from the potential difference and short-circuit current according to Ohm’s law.

### Determination of zonulin protein expression levels by western blot

LP or LPKM treated NCM460/MIMP samples were subjected to SDS-PAGE and transferred onto PVDF membranes. Membranes were incubated with the antibodies raised against zonulin (Lsbio) at a dilution of 1:100 for 2 h at room temperature, washed in TBS and then incubated for 1 h with corresponding HRP-conjugated secondary antibodies, and visualized using enhanced chemiluminescence.

### Detection of zonulin mRNA expression by quantitative real time PCR

Quantitative real time PCR (qRT-PCR) was used to determine the expression of Zonulin at the level of mRNA [17, 19]. Primers used in our study included:

forward primer, 5′-TCATCACGGCGCGCCAGG-3′

reverse primer, 5′-GGAGGTCTAGAATCTGCCCGAT-3′.

Total RNA was isolated from NCM460/MIMP cells using Trizol reagent (Invitrogen) followed by DNase I treatment. The quantity and quality of RNA was verified by determining the absorbance ratio at 260 and 280 nm, and by visualization of respective bands on agarose gels. For each sample, 600 ng mRNA was used in the reverse transcription reaction according to the manufacturer’s specifications (iScript kit, BioRad). mRNA was also detected by RT-PCR using a light-cycling system (LightCycler, Roche Diagnostics GmbH, Mannheim, Germany). The level of mRNA expression was expressed as the ratio of the mean reading of the experimental group over that of the control group for NCM460/MIMP cells.

### Verification of the zonulin pathway by examination of intestinal permeability *in vitro*

A zonulin overexpressing adenovirus was constructed as previously described [33]. Briefly, human zonulin cDNA was cloned into KpnI and XhoI sites of the pENTR 2B vector (Invitrogen), and then transferred to the pAd/CMV/V5-DEST vector (Invitrogen). The plasmids were linearized with PacI (Promega, Madison, WI) and transfected into 293A cells using Lipofectamine 2000. As a control, the pAd/CMV/V5-GW/lacZ vector (Invitrogen) was used to produce a lacZ-bearing adenovirus. NCM460 cells were transfected with Ad-zonulin or Ad-lacZ for 12 h. After transfection, the cells were washed with PBS and placed in fresh medium for western blot analysis and examination of intestinal permeability as described above.

### Clinical verification of zonulin pathway

It has been reported that postoperative septicemia is associated with bacterial translocation, which may be caused by the increase of intestinal permeability and barrier injury [2]. We used the postoperative septicemia to evaluate the relationship between human serum zonulin level and intestinal permeability [34]. 121 patients with colorectal cancer staged asT2-T3, N1, M0, according to the TNM staging system, were enrolled in this study. All patients underwent a radical colectomy at the Shanghai Sixth People’s Hospital, affiliated to Shanghai JiaoTong University in Shanghai or the Sixth Affiliated Hospital of Sun Yat-sen University in Guangzhou, between April 2009 and September 2012. The study design and protocols were reviewed and approved by the Human Research Review Committee in both the Shanghai Sixth People’s Hospital and the Sixth Affiliated Hospital of Sun Yat-sen University, and written informed consent for participation was obtained from each patient before their enrollment into the study. Serum samples were collected 1 day preoperatively,and 3 and 10 days after the surgical procedure. The concentrations of zonulin were determined using an ELISA kit, as previously described [35]. Briefly, plastic microtiter plates (Costar, Cambridge, MA) were coated with rabbit zonulin cross-reacting Zot derivative ΔG IgG antibodies (10 μg/ml in 0.1 mol/l sodium carbonate buffer, pH 9.0), which were generated as previously described [35]. After an overnight incubation at 4°C, plates were washed four times in TBS and blocked by incubation for 1 h at 37°C with TBS. After four washes, five ΔG serial standards (50, 25, 12.5, 6.2, 3.1, and 0 ng/ml) and patient sera samples (1:10 dilution in TBS) were added and incubated overnight at 4°C. After four washes with TBS + 0.2% Tween 20, plates were incubated with biotinylated anti-ZotIgG antibodies for 4 h at 4°C. A color reaction was developed using a commercial kit (ELISA amplification kit; Invitrogen). The absorbance at 495 nm was measured with a microplate auto-reader (Molecular Devices Thermomax Microplate Reader).

### Statistical analysis

The data were expressed as the mean ± standard deviation (SD) when normally distributed or as a median (range) when abnormally distributed. Statistical analyses were performed using the SPSS 13.0 software (SPSS Inc., Chicago, IL). SD between multiple groups was assumed to satisfy a normal distribution. Data were analyzed by one-way ANOVA when conditions of homogeneity of variance were present. P values <0.05 were considered to be statistically significant. Spearman’s correlation was used to assess the relationship between zonulin level and postoperative septicemia using SPSS 13.0.

## Results

### Loss of the MIMP sequence and expression in LPKM

**Figure 1:**
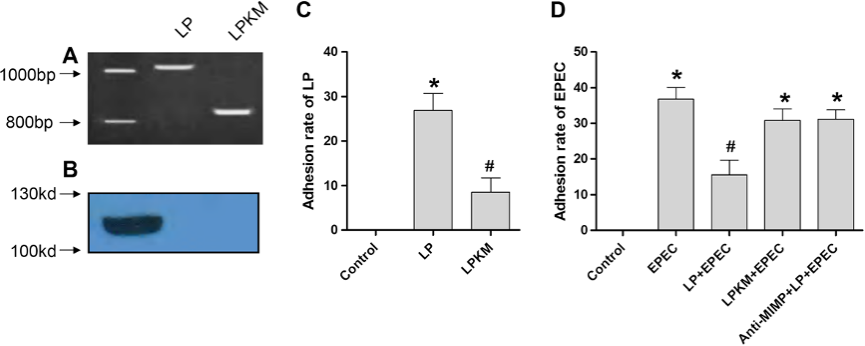
**Validation of the knockout of MIMP protein in LPKM and the adhesion of EPEC or LP to NCM460 cells. (A)** PCR amplicons over the integrated membrane protein region in LPKM confirmed the loss of ~ 200 bp in the genes surrounding the deletion; **(B)** Western blotting confirmed the non-expression of MIMP protein section in the whole bacteria protein of LPKM; **(C)** Adhesion of LP to NCM460 cells. The adhesion rate of LP to NCM460 cells was lower compared with the LPKM; **(D)** Adhesion of EPEC to NCM460/MIMP cells. The adhesion rate of EPEC to NCM460 cells was reduced when LP was added, while addition of LPKM had no effect about the inhibition of EPEC adhesion. However, the anti-MIMP deprived the effect of LP on the reduction of EPEC adhesion. *, P < 0.05, vs. Control; #, P < 0.05, vs. *group(s).

PCR and western blot were performed to confirm the deletion of MIMP gene for LPKM. PCR amplicons over the IMP region indicated that compared with LP of near 1000 bp, LPKM (about 800 bp) lost about 200 bp in the genes surrounding the deletion of LPKM (Figure 1A). Western blot showed a band of about 120 kd in the whole bacteria protein of LP butundetectable in the LPKM (Figure 1B).

### Decreased competitive inhibition of LPKM to EPEC adherence to NCM460 cells

The adhesion rate of LP to NCM460 cells was significantly lower compared with the LPKM (P < 0.001, Figure 1C). Detection of EPEC adherence indicated that the adhesion rate of EPEC to NCM460 cells was reduced significantly when LP was added, while adding LPKM had no effects on the inhibition of EPEC adhesion. However, the anti-MIMP deprived the effect of LP on the reduction of EPEC adhesion (P < 0.05, Figure 1D).

### LPKM had no effect on the EPEC-induced reduction of intestinal permeability

**Figure 2:**
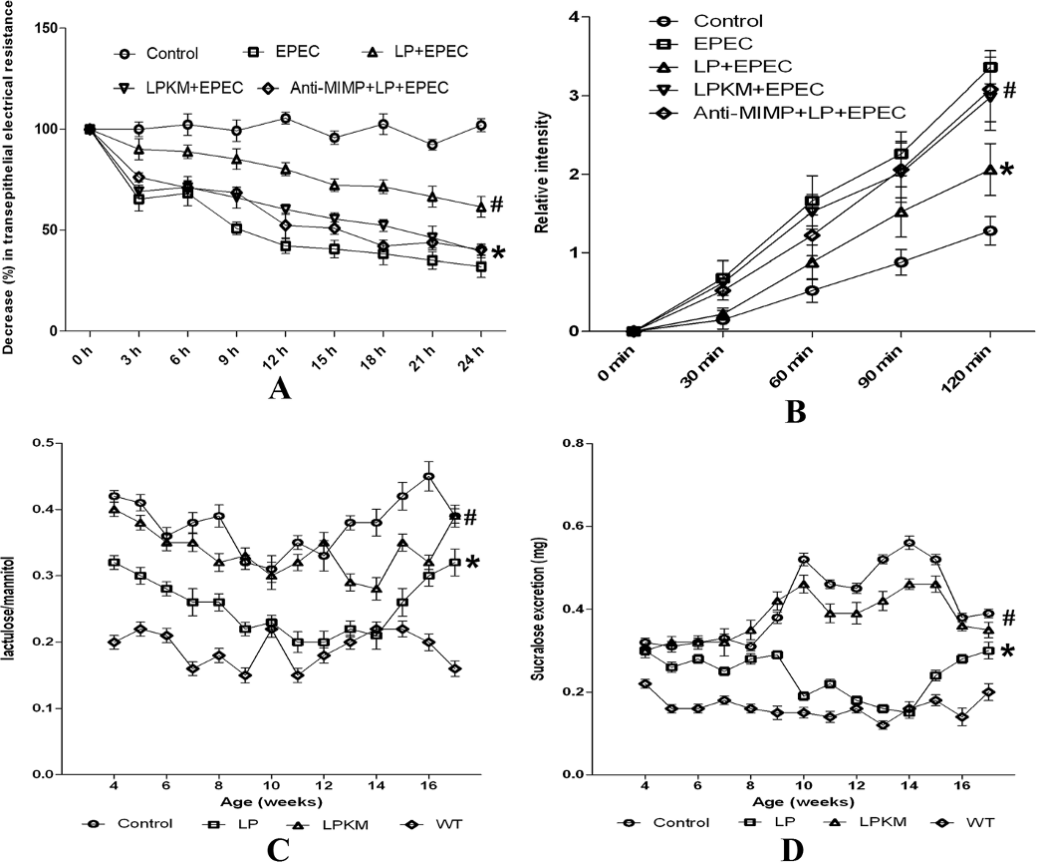
**Loss of effects of LPKM on the EPEC-induced reduction of intestinal permeability** ***in vitro*** **and** ***in vivo*** **. (A)** TER was found significantly decreased in the EPEC group, compared with control (P < 0.05), which was prevented by the simultaneous treatment of LP (P < 0.05). However, treatment with LPKM showed no effects on the EPEC-induced TER decrease (P > 0.05). Anti-MIMP antibody also inhibited the effect of LP on TER decrease (P > 0.05). **(B)** Similar findings were obtained for dextran permeability. **(C)** An increased small intestinal permeability was observed in the IL-10^-/-^ mice at age of 4 weeks and onwards, as compared with the wild-type mice (P < 0.05). LP could decrease small intestinal permeability in IL-10^-/-^ mice, and the effects were more evident at the age of 8–14 weeks when the small intestinal permeability returned to the normal level (P < 0.05). However, LPKM showed no decrease of small intestinal permeability in IL-10^-/-^ mice (P > 0.05). **(D)** IL-10^-/-^ mice had a significantly increased of colonic permeability, compared with control (P < 0.05). Treatment of LP had no significant effects on the intestinal permeability until week 10. At week 10–15, oral daily administration of pure milk containing LP effectively decreased colonic permeability of IL-10^-/-^ mice (P < 0.05). However, LPKM showed no help in decreasing colonic permeability of IL-10^-/-^ mice (P > 0.05). The intestinal epithelial monolayers were divided into five different experimental groups in triplicate. Each group used 10 animals for determination. *vs. EPEC group, P < 0.05; # vs. *, P < 0.05. The data were expressed as the mean ± standard deviation. Statistical analyses were performed using the SPSS 13.0 software (SPSS Inc., Chicago, IL). Data were analyzed by one-way ANOVA when conditions of homogeneity of variance were present.

TER in the NCM460 cell monolayers was found significantly decreased in response to EPEC infection, compared with uninfected control cells at 3–24 h (P < 0.05, Figure 2A). The EPEC-induced TER decrease was prevented by the simultaneous treatment of LP (P < 0.05, Figure 2A). However, treatment with LPKM showed a small effect on the EPEC-induced TER decrease (P > 0.05, Figure 2A). Anti-MIMP antibody also inhibited the effect of LP on EPEC-induced decrease in TER (P > 0.05, Figure 2A). Similar results were indicated for dextran permeability (Figure 2B).

The intestinal permeability and colonic damage *in vivo* were investigated using a sugar probes. Increased small intestinal permeability was observed in the IL-10^-/-^ mice at age of 4 weeks and onwards. Compared with the wild-type mice (P < 0.05, Figure 2C). Oral daily administration of pure milk containing LP for 4–17 weeks decreased small intestinal permeability in IL-10^-/-^ mice, and the effect was more evident at the age of 8–14 weeks when the small intestinal permeability returned to the normal level (P < 0.05, Figure 2C). However, administration of pure milk containing LPKM for 4–17 weeks showed no help in decreasing small intestinal permeability in IL-10^-/-^ mice (P > 0.05, Figure 2C). Meanwhile, IL-10^-/-^ mice had a significantly increased colonic permeability, compared with the wild-type mice (P < 0.05, Figure 2D). Treatment of LP had no significant effect on the intestinal permeability until week 10. At week 10–15, administration of pure milk containing LP was effective in decreasing colonic permeability in IL-10^-/-^ mice (P < 0.05, Figure 2D). However, administration of pure milk containing LPKM for 4–17 weeks showed no effect in decreasing colonic permeability in IL-10^-/-^ mice (P > 0.05, Figure 2D).

**Figure 3:**
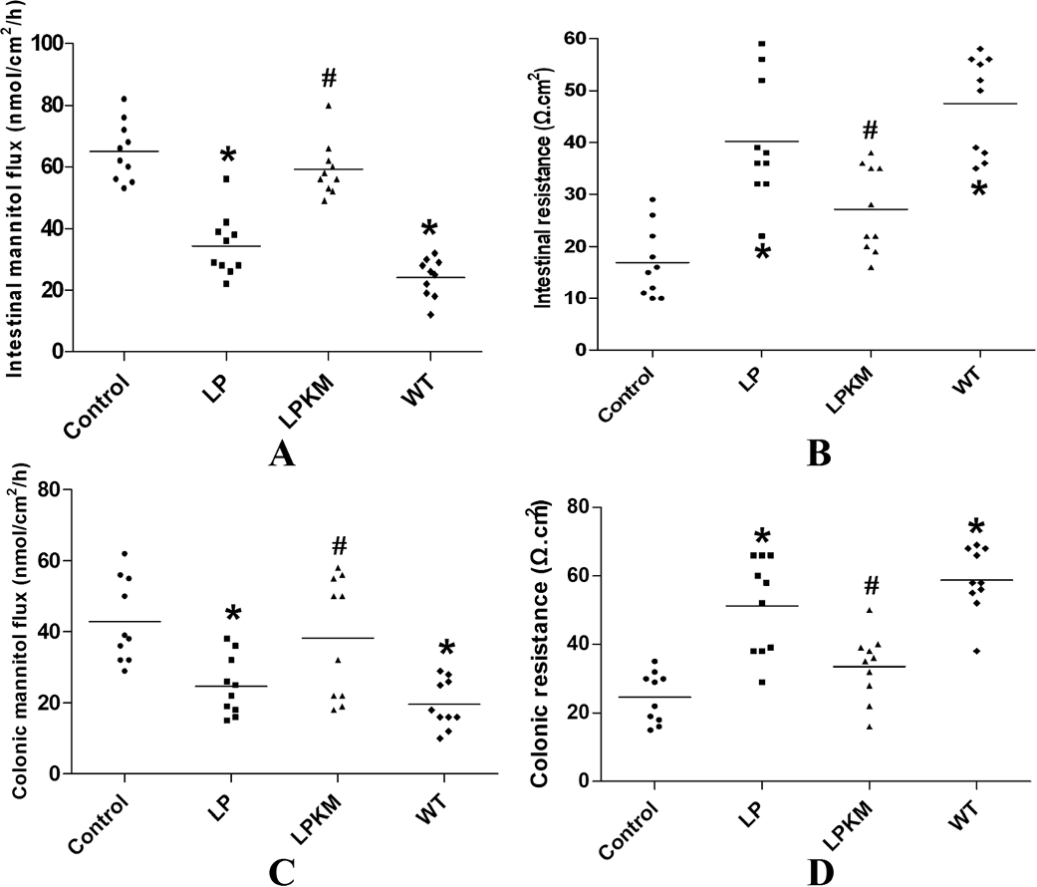
**Loss of alleviation of intestinal permeability of LPKM induced by EPEC** ***ex vivo*** **. (A)** The small intestinal permeability to mannitol in the IL-10^-/-^ mice was increased, compared with the wild-type group. In LP group, the small intestinal permeability to mannitol significantly decreased, compared with control (P < 0.05). However, in LPKM group, the small intestinal permeability to mannitol did not changed significantly (P > 0.05). **(B)** Similar results were indicated for intestinal resistance. **(C & D)** Similar results were observed in colonic tissues. Colonic permeability to mannitol increased in the IL-10^-/-^ mice with a corresponding decrease in TER, both of which were prevented by LP (P < 0.05). However, in LPKM group, colonic permeability to mannitol and TER did not changed significantly, compared with control (P > 0.05). Each group used 10 animals for determination. *vs. EPEC group, P < 0.05; # vs. *, P < 0.05. The data were expressed as the mean ± standard deviation. Statistical analyses were performed using the SPSS 13.0 software (SPSS Inc., Chicago, IL). Data were analyzed by one-way ANOVA when conditions of homogeneity of variance were present.

Ussing chamber assay was performed to evaluate the intestinal and colonic permeability in 8-week old mice tissues. The small intestinal permeability to mannitol in the IL-10^-/-^ mice was increased with a corresponding decrease in TER, compared with the wild-type group. In LP group, the small intestinal permeability to mannitol significantly decreased whereas TER significantly increased, as compared with the mice in control group (P < 0.05, Figure 3A and B). However, in LPKM group, the small intestinal permeability to mannitol and TER did not change significantly, compared with the mice in control group (P > 0.05, Figure 3A and B). Similar results were observed in colonic tissues. Colonic permeability to mannitol increased in the IL-10^-/-^ mice with a corresponding decrease in TER, both of which were prevented by not LPKM but LP (P < 0.05, Figure 3C and D)

### Loss of prevention of EPEC-induced increased zonulin expression level for LPKM

**Figure 4:**
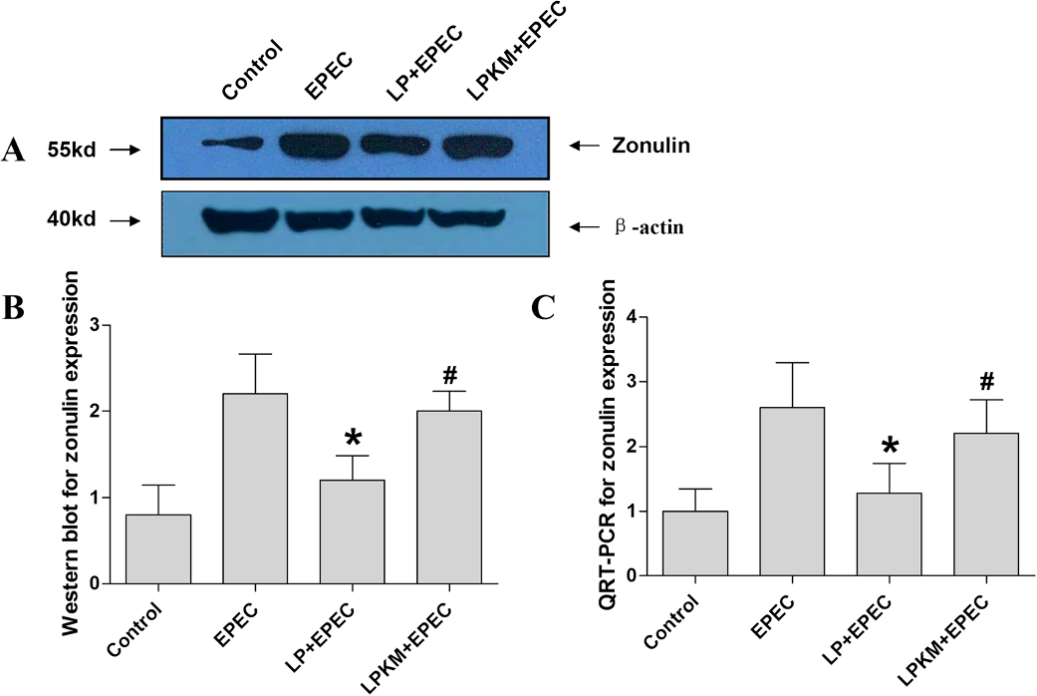
**Loss of prevention of EPEC-induced increased zonulin expression level for LPKM. (A)** Western blotting showed that zonulin protein expression level was higher in EPEC group, compared with the uninfected group. After the simultaneous treatment of LP, the increased zonulin protein expression level was lost. However, simultaneous treatment of LPKM did not change the increased zonulin protein expression level; **(B)** Semi-quantitative analysis of western blots results; **(C)** Detection of zonulin mRNA expression levels by qRT-PCR found that EPEC enhanced the zonulin protein expression level, which could not be prevented by LPKM but LP. *vs. EPEC group, P < 0.05; # vs. *, P < 0.05.

Semi-quantitative analysis of the western blots showed that zonulin protein expression level was higher in EPEC group, compared with the uninfected control group (P < 0.05, Figure 4A and B). After the simultaneous treatment of LP, the increased zonulin protein expression level was lost (P < 0.05, Figure 4A and B). However, simultaneous treatment of LPKM did not change the increased zonulin protein expression level (P > 0.05, Figure 4A and B). qRT-PCR also showed that EPEC enhanced the zonulin protein expression level (P < 0.05, Figure 4C), which could be prevented by not LPKM (P > 0.05, Figure 4C) but LP (P < 0.05, Figure 4C).

### Loss of reduced intestinal permeability for LP after overexpression of zonulin protein

**Figure 5:**
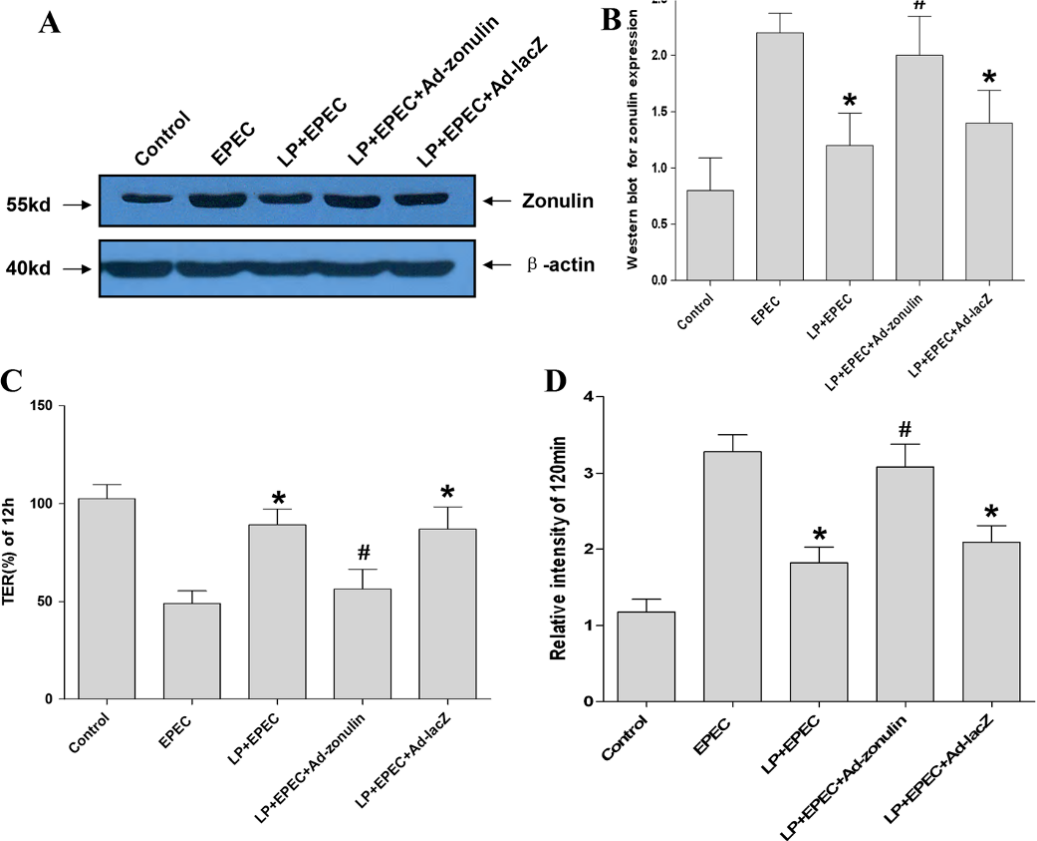
**LP lost the ability to reduce intestinal permeability after overexpression of zonulin protein in NCM460 cells. (A)** Zonulin protein expression level was higher in EPEC group, compared with the uninfected control group. After the simultaneous treatment of LP, the increased zonulin protein expression level was lost. The LP-induced increase level of zonulin was not changed after Ad-zonulin was transfected to NCM460 cells, while the increase was continue increased after Ad-lacZ was transfected; **(B)** Semi-quantitative analysis of western blots results; **(C)** TER in the NCM460 cell monolayers was found to be significantly decreased in response to infection with EPEC, as compared with uninfected control cells. The EPEC-induced change of TER was prevented by the simultaneous treatment of LP. However, the effects of LP to the EPEC induced change of intestinal permeability was inhibited with the transfection of Ad-zonulin to NCM460 cells, while that impaction did not existed when the Ad-lacZ was transfected instead of Ad-zonulin; **(D)** Dextran permeability was found to be significantly increased in response to infection with EPEC, as compared with uninfected control cells. The EPEC-induced change of dextran permeability was prevented by the simultaneous treatment of LP. However, the effects of LP to the EPEC induced change of intestinal permeability was inhibited with the transfection of Ad-zonulin to NCM460 cells, while that impaction did not existed when the Ad-lacZ was transfected instead of Ad-zonulin. *, P < 0.05, vs. LP + EPEC group; #, P < 0.05, vs. *.

Zonulin protein expression level was higher in EPEC group, compared with the uninfected control group (P < 0.05, Figure 5A and B). After the simultaneous treatment of LP, the increased zonulin protein expression level was lost (P < 0.05, Figure 5A and B). The LP-induced increase level of zonulin was not changed after Ad-zonulin was transfected to NCM460 cells (P > 0.05, Figure 5A and B), while the increase was continueing after Ad-lacZ was transfected (P < 0.05, Figure 5A and B).

TER in the NCM460 cell monolayers was found significantly decreased, and dextran permeability was found significantly increased in response to EPEC infection, as compared with uninfected control cells (P < 0.05, Figure 5C and D). The EPEC-induced change of TER and dextran permeability was prevented by the simultaneous treatment of LP (P < 0.05, Figure 5C and D). However, the effects of LP to the EPEC-induced change of intestinal permeability was inhibited with the transfection of Ad-zonulin to NCM460 cells (P > 0.05, Figure 5C and D), while the impact did not existed when Ad-lacZ was transfected instead (P < 0.05, Figure 5C and D).

### Relationship between zonulin level and postoperative septicemia

A direct correlation was found between the serum zonulin level and the postoperative septicemia (r = 1.000, P < 0.001).

## Discussion

In previous studies, we have identified and characterized MIMP as a domain of LP surface layer protein [8, 16], and it showed a key role in conferring the protection against pathogenic bacteria. A recent study found that knockout of the specific gene in lactobacillus could be a good model for investigating the mechanism of lactobacillus components [31]. Therefore, we established MIMP-knockout LPKM bacteria to investigate the mechanism of MIMP on the regulation of intestinal permeability in the present study. PCR amplicons over the integrated membrane protein region indicated the loss of ~ 200 bp in the genes surrounding the deletion of LPKM. Western blot confirmed that non-expression of MIMP protein section in the whole bacteria protein of LPKM. Our findings suggested that LPKM lost the MIMP sequence and did not expressed MIMP protein. It is proved that adhesion may be the first step of the interaction between lactobacillus and IECs, which could then exert its protective function against the intestinal barrier injury [27, 36, 37]. Our results indicated that after knockout of the MIMP [8, 16], LPKM lost the adhesive effects to NCM460 cells. EPEC adhesive assay confirmed that the competitively inhibitive effects of LPKM decreased significantly compared with LP, and showed similar effects in LP group added with anti-MIMP, which might be due to the loss of adhesive MIMP protein. To investigate the effects of LPKM on intestinal permeability, we performed the assay of TER, dextran permeability *in vitro*, sugar probe permeability *in vivo*, and Ussing chamber *ex vivo*. The intestinal permeability assay of NCM460 cells indicated that LPKM lost preventive effects on the EPEC-induced TER decrease and dextran permeability increase, which was similar to the role of anti-MIMP antibody [16]. Therefore, we deduce that the sequence of MIMP have an inhibitive effect on the increase of permeability of NCM460 cell monolayer. We also determined the intestinal permeability and colonic damage *in vivo* and *ex vivo* using the IL-10^-/-^ mice model, and found that LPKM lost the ability to lower both the small intestinal and colonic permeability of mice, as the permeability to mannitol and TER did not change significantly both in intestinal and colonic tissues. Importantly, our results suggested that LPKM lost the effects on the intestinal and colonic permeability, and MIMP might have inhibitive effects on the increase of intestinal and colonic permeability. Because it has been reported that the reduction of small intestinal permeability could attenuate the colitis and protect the intestinal barrier function in the IL-10^-/-^ mice [16, 32], MIMP may alleviate the intestinal inflammation by reducing small intestinal and colonic permeability.

Zonulin is a recent discovered protein that participates in TJ between IECs in the digestive tract [19]. Zonulin was originally discovered as the target of zonula occludens toxin, which is secreted by cholera pathogen Vibrio cholerae [38]. It has been reported as a marker of the increased gut permeability in coeliac disease [20] and type 1diabetes mellitus [35]. High expression of zonulin could reflect the increase of intestinal permeability [22]. However, the relationship between probiotics and zonulin protein remains uninvestigated. The molecular mechanism underlying how LP can exert its effects on intestinal permeability has still not been clarified. Therefore, we promote our hypothesis that LP may exert the regulative effects on intestinal permeability via the zonulin pathway. Results indicated that LP could inhibit the EPEC-induced increase of zonulin expression, while LPKM could not, which suggested that MIMP may lower the intestinal permeability by inhibiting the expression of zonulin in IECs, and then alleviate the intestinal inflammation and protect the normal intestinal barrier function. To verify the zonulin pathway, during the interaction between LP and the intestinal permeability, the zonulin overexpressing adenovirus was constructed and transfected into the NCM460 cells. Results indicated that overexpression of zonulin protein deprived the reduced effects of LP to intestinal permeability, including the increase of TER and decrease of macro molecular dextran permeability. Furthermore, we evaluated the correlation between serum zonulin levels and postoperative septicemia, which was found positive. In this study, we first verified the critical role of zonulin in the regulation of intestinal permeability.

Above all, MIMP might be an important protein with protective effects on intestinal barrier, which could be used as a new drug to prevent and treat intestinal barrier dysfunction [2, 7, 17]. Since lactobacillus may have a risk of translocation and could not be used combined with antibiotics, MIMP will show its own advantages [17, 39, 40]. Additionally, zonulin could also be used as a biomarker of intestinal dysfunction [41].

One limitation of our study is that we did not have a verification of zonulin using the human serum samples after administration of LP. And this test is now in progress in our hospital. Furthermore, recently, some studies on barrier function mediated by newly discovered molecules or cells of IEC itself are drawing more attention [42], such as intestinal villi brush border alkaline phosphatase (IAP) [43–45], intracytoplasmic protein phosphatase 2A (PP2A) [46, 47], their interaction with MIMP should be further investigated in the following study [40].

## Conclusion

MIMP-knockout LPKM lost the protective effects against the injury of IECs, therefore, MIMP might have protective effects on the intestinal epithelial cells associated with the zonulin pathway.

## Authors’ information

Co-first authors: Zhihua Liu, Liang Kang, Chao Li and Chao Tong.
